# Tele-neurosurgery: Expanding access and expertise through remote consultation and virtual surgery

**DOI:** 10.1016/j.bas.2024.102920

**Published:** 2024-08-08

**Authors:** Vanitha Marunganathan, Ajay Guru

**Affiliations:** Department of Cariology, Saveetha Dental College and Hospital, Saveetha Institute of Medical and Technical Sciences, Saveetha University, Chennai, 600077, Tamil Nadu, India

Dear Editor,

In the evolving landscape of neurosurgery, the integration of telemedicine technologies presents a transformative opportunity to expand access to specialized care and expertise globally. This innovative application of telemedicine allows experienced neurosurgeons to remotely consult and collaborate with local surgical teams, particularly in underserved regions where access to such expertise is limited. Initiatives like "virtual surgical mentorships" enable remote guidance during complex procedures, thereby enhancing the quality of care and providing an invaluable learning platform for local surgeons (Cannizzaro et al., 2022). The integration of virtual reality (VR) and augmented reality (AR) technologies further enhances this field by enabling immersive preoperative planning and providing real-time data overlays during surgeries. These tools not only improve surgical precision and situational awareness but also offer new opportunities for education and training in complex neurosurgical procedures (Seeliger et al., 2022).

Artificial intelligence (AI) plays a critical role in advancing tele-neurosurgery by providing real-time decision support and analysis (Sudhakaran, 2024). AI algorithms can monitor surgical procedures, detect anomalies, and predict potential complications, thus offering timely interventions and enhancing patient safety (Fernandes, 2021). The development of a global network of neurosurgical centers equipped with advanced telemedicine capabilities is essential to maximize the benefits of tele-neurosurgery. Such a network would facilitate the exchange of knowledge and resources, support international clinical trials, and ensure that high standards of care are maintained across different regions (Xu et al., 2022). Moreover, this global collaboration could accelerate the development and dissemination of innovative neurosurgical techniques and technologies.

However, the adoption of tele-neurosurgery must be approached with careful consideration of ethical and logistical challenges. Ensuring patient privacy, managing the legal implications of cross-border medical consultations, and maintaining consistent quality of care are paramount concerns. Establishing standardized protocols and guidelines is crucial to address these issues and to ensure the responsible and effective use of tele-neurosurgical technologies (Solimini et al., 2021). By fostering innovation and collaboration, we can leverage these advancements to improve access to high-quality neurosurgical care worldwide.

## Declaration of competing interest

The authors declare that they have no known competing financial interests or personal relationships that could have appeared to influence the work reported in this paper.
